# Essential role of HCMV deubiquitinase in promoting oncogenesis by targeting anti-viral innate immune signaling pathways

**DOI:** 10.1038/cddis.2017.461

**Published:** 2017-10-05

**Authors:** Puja Kumari, Irene Saha, Athira Narayanan, Sathish Narayanan, Akinori Takaoka, Nachimuthu Senthil Kumar, Prafullakumar Tailor, Himanshu Kumar

**Affiliations:** 1 Department of Biological Sciences, Laboratory of Immunology and Infectious Disease Biology, Indian Institute of Science Education and Research (IISER) Bhopal, Bhopal 462066, India; 2 Laboratory of Innate Immunity, National Institute of Immunology (NII), New Delhi 110067, India; 3 Department of Biological Sciences, Laboratory of Virology, Indian Institute of Science Education and Research (IISER) Bhopal, Bhopal 462066, India; 4 Division of Signaling in Cancer and Immunology, Institute for Genetic Medicine, Hokkaido University, Sapporo, Japan; 5 Department of Biotechnology, Mizoram University, Aizawl 796004, India; 6 Laboratory of Host Defense, WPI Immunology, Frontier Research Centre, Osaka University, Osaka 5650871, Japan

## Abstract

Cancer is a multifactorial disease and virus-mediated carcinogenesis is one of the crucial factors, which is poorly understood. Human cytomegalovirus (HCMV) is a herpesvirus and its components have been evidenced to be associated with cancer of different tissue origin. However, its role in cancer remains unknown. Here, we identified a conserved herpesviral tegument protein known as pUL48 of HCMV, encoding deubiquitinase enzyme, as having a key role in carcinogenesis. We show using deubiquitinase sufficient- and deficient-HCMV that HCMV deubiquitinase is a key in inducing enhanced cellular metabolic activity through upregulation of several anti-apoptotic genes and downregulation of several pro-apoptotic genes expression. Furthermore, HCMV deubiquitinase acquires pro-tumor functions by inhibiting PRR-mediated type I interferon via deubiquitination of TRAF6, TRAF3, IRAK1, IRF7 and STING. Taken together, our results suggest that HCMV infection may promote oncogenesis by inhibiting innate immunity of the host.

Cancer is a multifactorial disease causing death worldwide and proves to be a burden on human health. DNA viruses such as Epstein–Barr Virus, Hepatitis B Virus, Human Papilloma Virus and Kaposi’s Sarcoma-Associated Herpesvirus cause a wide range of malignancies such as nasopharyngeal carcinoma, Burkitt’s lymphoma, head and neck cancer, and cervical cancer in the host.^1, 2^ In addition, RNA viruses such as Hepatitis C Virus, Human Mammary Tumor Virus, Torque Teno Virus and Human Endogenous Retrovirus also acquire different strategies for oncogenesis.^1^ A growing list of cancer-associated viruses indicates that viral infection can directly or indirectly bring upon carcinogenic state. Human cytomegalovirus (HCMV) is among those DNA viruses that have been found associated with cancers.^3, 4^ However, HCMV has not been characterized as an oncogenic virus. HCMV structural components have been found in tumor tissues^3, 4, 5^ and reported to be involved in promoting a favorable microenvironment for oncogenic transformation of infected cells.^6, 7^ HCMV infects 90% of the world’s population, hence, studying the carcinogenic potential of HCMV and understanding its molecular mechanism may help stop progression of various cancers. Although, HCMV infection is mostly asymptomatic due to host anti-viral immunity, it may lead to oncogenic transformation of normal cells and cancer, when host is immunocompromised owing to immunosuppressive drugs or infection with HIV.

Numerous studies have shown that type I interferons (I-IFNs) have a key role in inhibition of cancer.^8, 9, 10, 11^ Virus-infected cells undergo apoptosis as a defense against spread of infection. However, HCMV acquires several strategies to inhibit apoptotic pathway and establish a successful infection.^3^ These survival strategies acquired by HCMV can lead to uncontrolled cell growth.

Here, we identified a novel role of HCMV deubiqutinase (DUB) in oncogenesis. HCMV-DUB is encoded by unique long48 (UL48) gene, a conserved high-molecular-weight protein across the herpesviruses. We have found that HCMV-DUB inhibits expression of various pro-apoptotic genes and induces expression of anti-apoptotic genes. HCMV-DUB enables cells to surpass the G1-phase rapidly and enter into other phases of cell cycle required for cell division. Cellular DUBs have an important role in many signaling pathways, including immune signaling, apoptosis, oncogenesis and developmental pathways.^12, 13, 14^ Likewise, our findings as well reveal that upon infection, HCMV-DUB inhibits synthesis of I-IFNs, an anti-cancer factor, by deubiquitinating several signaling molecules such as TNF receptor-associated factor (TRAF)-6 and -3, interleukin-1 receptor-associated kinase-1 (IRAK1), interferon regulatory factor (IRF)-7 or stimulator of interferon genes (STING) that have a key role in anti-viral innate immunity. Inhibition of I-IFNs by HCMV-DUB correlates with decreased expression of several pro-apoptotic genes and increased expression of anti-apoptotic genes, which also indicates its oncogenic potential during infection.

## Results

### HCMV induces oncogenic properties

**Figure 1:**
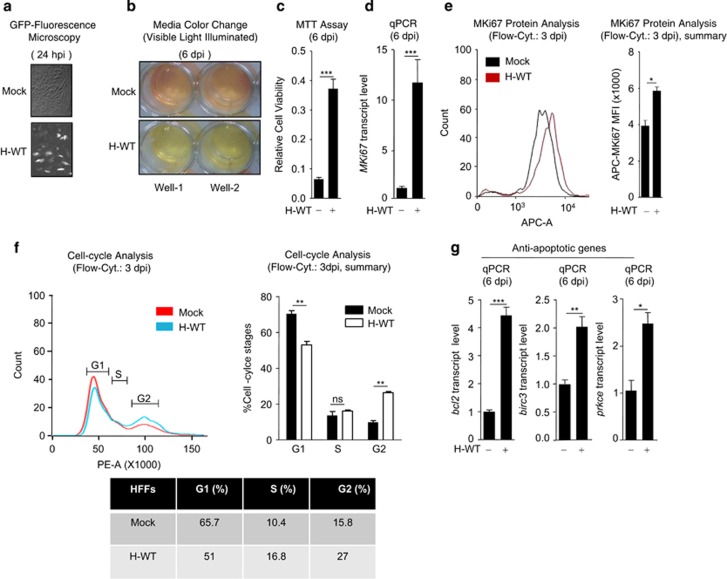
HCMV infection induces oncogenic properties in non-transformed HFFs. (**a**) GFP expression was observed under the fluorescence microscope at 24 h post infection (hpi) with GFP-tagged WT-HCMV (H-WT) in HFFs. (**b**) Culture media color change (red to yellow) was visually observed on day 6 post infection (dpi), in HFFs infected with H-WT. (**c**) Cell viability (metabolic activity) of mock or H-WT-infected HFFs was quantified by MTT assay, on 6 dpi. (**d**) Transcript and (**e**) protein of *MKi67* was quantified by qPCR and flow cytometer respectively, in mock, H-WT-infected HFFs, (**f**, left, right, bottom) flowcytometry was done for mock and H-WT-infected HFFs by staining them with propidium iodide (PI, shown in *x* axis) on 3 dpi, to detect cell cycle stages upon HCMV infection and (**g**) Anti-apoptotic gene (*bcl2*, *birc3* and *prkce*) status was compared by qPCR in mock or H-WT infected HFFs on 6 dpi. Shown results are the representative of three (**a**–**d**, **g**) or two (**e**, **f**) independent experiments. (**f**) Statistical analysis was done with data of two independent experiments. Differences were considered statistically significant with a *P-value<0.05, **P-value<0.01 and ***P-value<0.001, ns, non-significant difference (*P*-value>0.05)

Association of HCMV antigens with various cancer types is well known. However, whether HCMV promotes cancer upon infection is unknown. To investigate the role of HCMV in oncogenesis, non-transformed human foreskin fibroblasts (HFFs) were infected with a GFP-tagged laboratory strain of wild-type HCMV (WT-HCMV), AD169 (MOI 5). On second day post infection (dpi), virus infection was observed by GFP fluorescence (Figure 1a). On sixth dpi, infected cells showed a characteristic cytopathic effect (CPE) and also change in growth media color (red to yellow) (Figure 1b). The color change owing at least in part to release of marker GFP from infected cells but also may indicate enhanced metabolic activity during infection. To evaluate the possibility more directly, we performed an MTT assay comparing infected cells with uninfected. A fourfold higher metabolic activity was observed for WT-HCMV-infected cells compared with uninfected cells (Figure 1c). In addition, an enhanced level of RNA and protein of *MKi67*, a cell proliferation marker gene, was observed in WT-HCMV-infected HFFs compared with uninfected HFFs (Figures 1d and e), consistent with the result of MTT assay. This led us to further investigate the cell cycle stages of HCMV-infected cells by flowcytometry, which showed a detectable skewing pattern toward G2-phase in infected cells than in uninfected cells (Figure 1f).

The majority of viral infection leads to cell death, as a protective innate immune response to reduce viral-load within the host. The cell death is triggered through activation of apoptotic pathways, however, HCMV infection increased cell survival via significantly enhancing the expression of anti-apoptotic genes such as *bcl2*, *birc3* and *prkce*, compared with mock infection (Figure 1g), indicating that HCMV infection protects cells from apoptosis and promotes cell survival. During cancer progression, expression of anti- and pro-apoptotic genes is modulated to support cancer progression. Most often increase in expression of anti-apoptotic genes and decrease in expression of pro-apoptotic genes favor cancer progression. Collectively, these results indicate that HCMV infection induces oncogenic properties in non-transformed cells through upregulation of anti-apoptotic genes, resulting into enhanced cell proliferation.

### DUB activity of HCMV-pUL48 induces oncogenic properties

**Figure 2:**
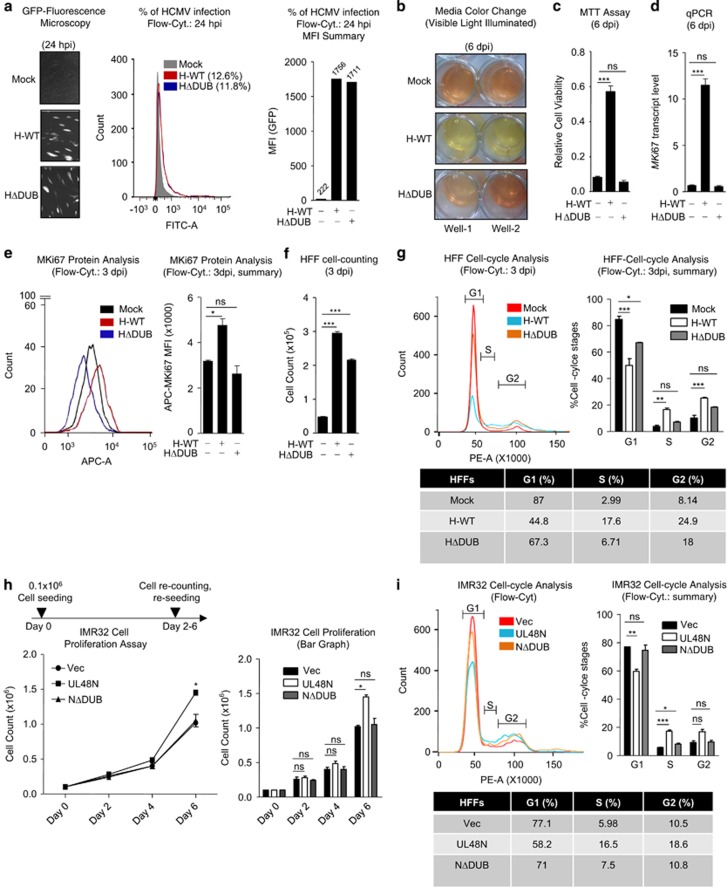
HCMV deubiquitinase is responsible for induction of oncogenic properties. (**a**) HFFs were infected with equal MOI of GFP-tagged H-WT and HΔDUB; equal amount of GFP expression was microscopically observed at 24 h post infection (hpi) with GFP-tagged H-WT and HΔDUB in HFFs. Quantification of GFP fluorescence (HCMV infection) was done with flow cytometer; histogram shows the percentage of HCMV infection at 24 hpi, measured by flowcytometry and bar- graph shows the GFP-Mean Fluorescent Intensity (MFI) of the respective histograms. (**b**) Culture media color change (red to yellow) was visually observed on 6 dpi, in HFFs infected with H-WT. (**c**) Cell viability (metabolic activity) was quantified and compared among mock, H-WT and HΔDUB-infected HFFs, by MTT assay. (**d**) Transcript of proliferation marker gene *MKi67* was quantified by qPCR in mock, H-WT and HΔDUB-infected HFFs. (**e**, left) MKi67 protein analysis was done in mock, H-WT and HDUB-infected HFFs by flowcytometry (**e**, right) MFI was calculated for respective MKi67 histograms. (**f**) Proliferation rate of Mock, H-WT and HΔDUB-HCMV-infected HFFs was analyzed by seeding them into equal number, harvesting them on 3 dpi and counting them to compare the total number of cells. (**g**, left, right, bottom) Cell Cycle stages of mock, H-WT and HΔDUB-infected HFFs were analyzed by flowcytometry by staining these HFFs with propidium iodide (PI) (shown in *x* axis) on 3 dpi. (**h**, left, right) The cell proliferation rate of IMR32 cells, stably expressing Vec, UL48N and UL48NΔDUB, was compared by initially seeding them into a density of 0.1 × 10^6^ cells, followed by counting and re-seeding them on every alternate day for 6 days. (**i**, left, right) Cell Cycle stages IMR32 stably expressing vector UL48N or NΔDUB were analyzed by flowcytometry by staining the cells with PI. dpi: days post infection, Vec: Empty Vector, NΔDUB: UL48NΔDUB. Shown results are the representative of three (**a**–**d**, **f**, **h**, **i**) or two (**e**, **g**) independent experiments. (**g**, **h**) Statistical analysis was done with data of two (**g**) and three (**h**) independent experiments and the difference was calculated between mock *versus* WT-HCMV infection or mock *versus* HΔDUB-HCMV infection (**g**) or between Vec *versus* UL48N or Vec *versus* NΔDUB (**h**). Differences were considered statistically significant with a *P-value<0.05, **P-value<0.01 and ***P-value<0.001, ns, non-significant difference (*P*-value>0.05)

Previously, it has been reported that the inactive DUB of Marek’s disease herpesvirus compromises the ability of that virus to cause lymphoma in chickens.^15^ In addition, reports suggest that deregulation of cellular DUBs could also promote various cancer types in humans and mouse.^16, 17, 18^ However, HCMV-DUB and its role in oncogenesis are not known. We hypothesized that HCMV-DUB may also have such a role. To test this, we used a previously reported HCMV mutant virus that encodes an inactive DUB.^19^ After infecting HFFs with equal amount of WT and DUB-mutant (ΔDUB) GFP-tagged HCMV, an equal amount of virus infection (GFP Fluorescence) was observed on second dpi (Figure 2a). On sixth dpi, ΔDUB-HCMV-infected cells showed much less-metabolic activity than WT-HCMV-infected cells, however, comparable to mock infected cells (Figure 2b). To confirm the differential responses of WT- and ΔDUB-HCMV, MTT assay and *MKi*67 transcript and protein analysis were performed for cell metabolic activity and cell proliferation, respectively, which indicated that ΔDUB-HCMV reduced cell metabolic activity and proliferation compared with WT-HCMV (Figures 2c–e). Furthermore, HFF cell counting and cell cycle analysis after three dpi with HCMV, revealed that WT-HCMV infection causes enhanced cell proliferation (Figure 2f) and more cell accumulation in the G2-phase compared with mock or ΔDUB-HCMV infection (Figure 2g).

Next, to confirm that HCMV-DUB can stimulate cell proliferation, we created as previously reported a mammalian expression plasmid,^20^ encoding amino acid 1–1162 of pUL48, a functional DUB domain (UL48N) and UL48N lacking DUB function (UL48NΔDUB) of HCMV. The IMR32 cells (a non-invasive neuroblastoma cell line) stably expressing UL48N, UL48NΔDUB or empty vector (Vec) were generated and subjected to cell proliferation analysis from Day 0 to Day 6 after seeding at a density of 0.1 × 10^6^ cells and by counting the number of cells on every alternate day. The IMR32 cells, stably expressing WT-UL48N showed rapid proliferation than those stably expressing either ΔDUB-UL48N or Vec (Figure 2h). This was further confirmed by cell cycle analysis of IMR32 cells, which showed an enhanced accumulation of UL48N expressing IMR32 cells in S- and G2-phases compared with Vec and UL48NΔDUB (Figure 2i). Collectively, these results indicate that HCMV-DUB can induce cell proliferation and UL48-DUB is crucial for this activity.

### HCMV-DUB induces cancer hallmarks

**Figure 3:**
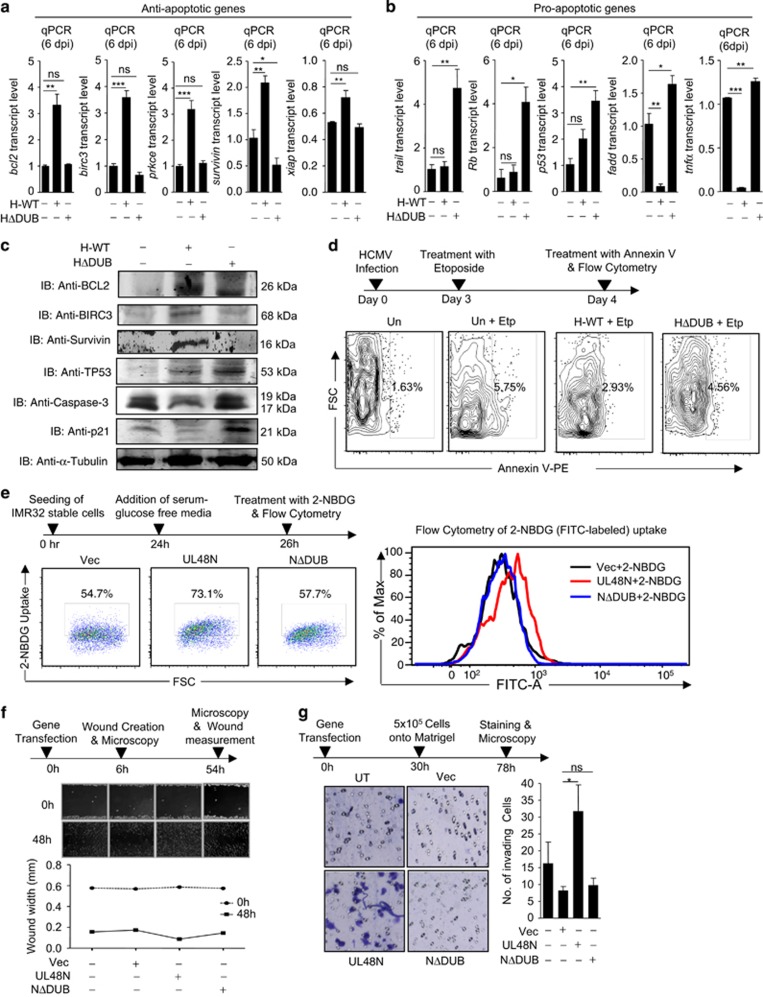
HCMV deubiquitinase induces cancer hallmarks: (**a**, **b**) HFFs were infected with equal MOI of GFP-tagged WT-HCMV (H-WT) and ΔDUB-HCMV (HΔDUB) and analyzed for apoptosis-associated genes on 6 dpi, (**a**) transcript level of anti-apoptotic genes *bcl2*, *birc3*, *prkce, survivin* and *xiap* as well as (**b**) transcript level of pro-apoptotic genes *trail*, *Rb*, *p53*, *fadd* and *tnfα* was measured by qPCR in mock, H-WT and HΔDUB-infected HFFs. (**c**) Endogenous protein level of BCL2, BIRC3, Survivin, TP53, Caspase-3 and p21 was analyzed in HFFs upon 8 dpi with H-WT and HΔDUB (**d**) HCMV-infected HFF cells were treated with 30*μ*M etoposide on 3 dpi and analyzed for apoptosis rescue 24 h later by quantifying Annexin V level through flow cytometer. (**e**, left, right) IMR32 cells stably expressing Vec, UL48N and UL48NΔDUB were glucose starved for 2 h and treated with FITC-labeled Glucose for 10 min and then subjected to flow cytometer to observe the glucose uptake (**f**, top) Wound was created in IMR32 cells transiently transfected with Vec, UL48N and UL48NΔDUB expression plasmids as depicted and healing was observed for 48 h, (**f**, bottom) wound size was measured. (**g**, left) 5 × 10^5^ IMR32 cells transiently transfected with Vec, UL48N and UL48NΔDUB expression plasmids were resuspended in serum deprived growth media and inoculated onto the matrigel layer containing insert, which was further kept in contact with serum containing growth media. Tissue invasion of these IMR32 cells was analyzed through matrigel invasion. (**g**, right) Matrigel invaded cells were counted and graph was plotted. Blue dots represent the invaded cells. Vec: Empty Vector, dpi: days post infection, NΔDUB: UL48NΔDUB, Etp: Etoposide, FITC-A: FITC-Area. Shown results are the representative of three (**a**, **b**, **d**, **e**, **f**, **g**) or single (**c**) independent experiments. Differences were considered statistically significant with a *P-value<0.05, **P-value<0.01 and ***P-value<0.001, ns, non-significant difference (*P*-value>0.05). See also Supplementary Figure S1a–S1l

The majority of viral infections lead to cell death through apoptotic pathways, which is a protective innate immune response. To understand whether HCMV-DUB modulates pro-apoptotic and anti-apoptotic gene expressions during infection, WT-HCMV and ΔDUB-HCMV-infected HFFs were first analyzed for the expression of anti- and pro-apoptotic genes through qPCR, on 6th dpi. Transcription of anti-apoptotic genes such as *bcl2, birc3*, *prkce*, *survivin* and *xiap* was significantly increased by many folds, in WT-HCMV-infected HFFs, however the transcript level remained either unchanged or reduced in ΔDUB-HCMV-infected HFFs compared with mock (Figure 3a). Furthermore, protein-expression analysis of anti-apoptotic genes–*bcl2*, *birc3* and *survivin*–showed an increased expression in WT-HCMV-infected HFFs than in mock or ΔDUB-HCMV-infected HFFs (Figure 3c). Expression level of few other anti-apoptotic genes such as *ciap1*, *cflip and mcl-1* decreased in case of both, WT and ΔDUB-HCMV infection compared with mock infection (Supplementary Figures S1a–S1c), which suggests HCMV-DUB has no specific role in regulating these anti-apoptotic genes. Surprisingly, transcript level of *bcl-xl* anti-apoptotic gene was reduced owing to WT-HCMV infection, whereas remained unchanged upon ΔDUB-HCMV infection (Supplementary Figure S1d). Similarly, transcription of pro-apoptotic genes such as *trail*, *Rb*, *p53*, *fadd* and *tnfα*, remained either unchanged, less induced or reduced in WT-HCMV-infected cells compared with mock, whereas expression of these pro-apoptotic genes was higher in the ΔDUB-HCMV-infected cells (Figure 3b). The p53 protein was induced upon infection with both WT- and ΔDUB-HCMV, however induction was comparatively less in WT-HCMV-infected HFFs (Figure 3c). Transcription of few other pro-apoptotic genes such as *bad* and *bax* was decreased upon infection with both, WT- and ΔDUB-HCMV, suggesting no specific role of HCMV-DUB in regulating *bad* and *bax*, whereas transcription of *caspase-8,* and *p21* was reduced only in WT-HCMV-infected HFFs, and remained unchanged in ΔDUB-HCMV-infected HFFs (Supplementary Figures S1f–S1i). Furthermore, protein analysis of p21 and caspase-3 showed a decreased expression in WT-HCMV-infected cells compared with mock (Figure 3c). The expression of several other anti- and pro-apoptotic genes was tested, with no evidence of differences between WT- and ΔDUB-HCMV-infected cells compared with mock (Supplementary Figures S1e, S1j–S1l) suggesting no involvement of HCMV infection to regulation of these genes. Differential expression of anti- and pro-apoptotic genes during WT-HCMV- and ΔDUB-HCMV infection indicates that HCMV-pUL48 DUB activity interferes with the induction of apoptosis and may thus induce oncogenic properties. Consistent with this interpretation, we found that the ability of anti-cancer drug etoposide to promote apoptosis, was reduced by WT-HCMV infection but not by ΔDUB-HCMV infection (Figure 3d).

Next, we tested uptake of glucose, as cancer cells require increased glucose for metabolism and cell division.^21^ To this end, IMR32 cells stably expressing Vec, UL48N and UL48NΔDUB were glucose starved and treated with FITC-labeled glucose (2-NBDG). Flow cytometric analysis revealed that cells expressing UL48N took-up more 2-NBDG than those expressing Vec or UL48NΔDUB (Figure 3e). Moreover, overexpression of UL48N, increased the cell migration (Figure 3f) and tissue invasion (Figure 3g) of IMR32 cells (tested by wound-healing and matrigel invasion assay, respectively), whereas UL48NΔDUB did not induce any of these characteristics of cancer. These data suggest that HCMV-DUB could promote several oncogenic properties and possibly initiate or promote cancer.

### HCMV-DUB inhibits anti-viral innate immunity for oncogenesis

**Figure 4:**
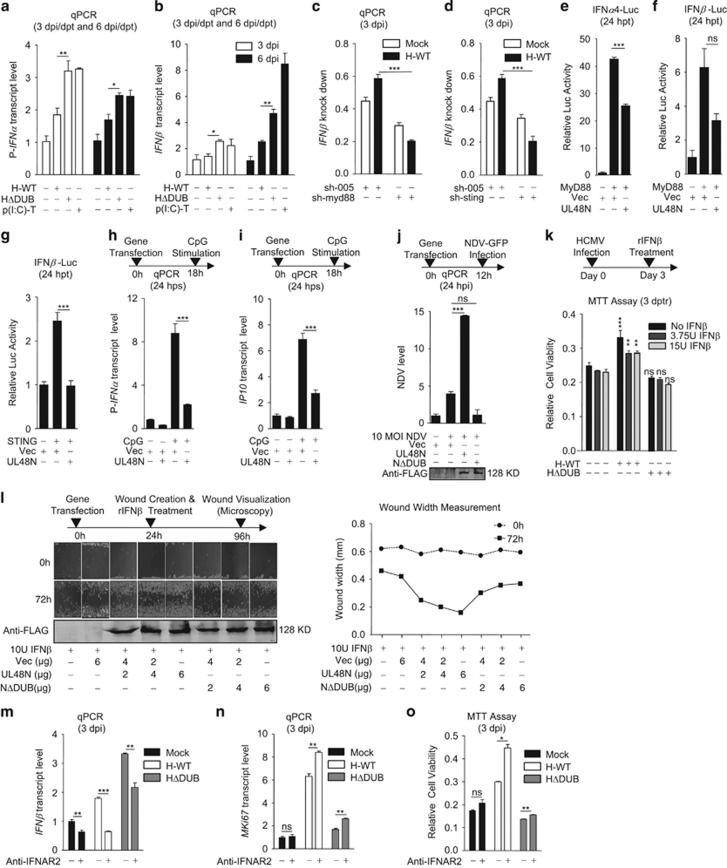
HCMV deubiquitinase inhibits anti-viral innate immunity to induce oncogenesis (**a**) Transcript of pan-interferon-*α* (P*-IFNα)* and (**b**) *IFNβ* was measured by qPCR upon WT-HCMV (H-WT) and ΔDUB-HCMV (HΔDUB) infection or poly(I:C) transfection to HFF cells, on 3 and on 6 dpi or dpt, as indicated. (**c, d**) Transcript of *IFNβ* was measured by qPCR upon H-WT infection to myd88 (**c**) or sting (**d**) knocked down HFFs. (**e–g**) Luciferase assay was done for *IFNα4* and *IFNβ* promoters in HEK293 cells, as depicted. (**h, i**) Transcript level of P-*IFNα* and *IP10* was measured in HeLa cells transfected with UL48N and stimulated with CpG. (**j**, top) NDV transcript level was measured in IMR32 cells transiently transfected with Vec, UL48N and UL48NΔDUB expression plasmids as depicted. (**j**, bottom) Overexpression of UL48N and UL48NΔDUB was detected by using anti-FLAG antibody. (**k**) MTT assay was done with HCMV-infected and rIFN-*β* treated HFFs as depicted and significance of WT- or HΔDUB infection group was calculated compared with mock. (**l**, left-top) Wound-healing was observed in IMR32 cells transiently transfected with increasing amount of Vec, UL48N and UL48NΔDUB expression plasmids and treated with constant amount of rIFN-β (10 U), as depicted, (**l**, right) wound size was measured and (**l**, left bottom) expression of UL48N and UL48NΔDUB was analyzed using anti-FLAG antibody. (**m, n**) Transcript of *IFNβ* and *MKi67* was measured by qPCR upon anti-IFNAR2 antibody treatment and WT-HCMV (H-WT) or ΔDUB-HCMV (HΔDUB) infection to HFF cells, on 3 dpi as indicated. (**o**) MTT assay was done with HFF cells upon anti-IFNAR2 antibody treatment (for 4 h) and WT-HCMV (H-WT) or ΔDUB-HCMV (HΔDUB) infection to HFF cells, on 3 dpi as indicated. Vec: Empty Vector, NΔDUB: UL48NΔDUB, p(I:C)-T: Polyinosinic:polycytidylic-Transfection, dpi: days post infection, dpt: days post transfection, hpt: hours post transfection, hps: hours post-stimulation, hpi: hours post infection, dptr: days post treatment, sh: short-hairpin, rIFN: recombinant interferon, IFNAR: Type I interferon *α/β* Receptor. Shown results are the representative of two (**a**–**d**, and **h**–**o**) or three (**e–g**) independent experiments. Differences were considered statistically significant with a *P-value<0.05, **P-value<0.01 and ***P-value<0.001, ns, non-significant difference (*P*-value>0.05). See also Supplementary Figure S2a–S2e

Viral infections can induce cellular anti-viral innate immune responses, including I-IFNs, responsive to pattern recognition receptors (PRRs). Besides anti-viral immunity, the I-IFNs are also known to have a key role in elimination of self-altered or tumor cells through induction of pro-apoptotic genes such as *p53*,^22^ *Rb*,^23, 24^ *trail*^10, 25^ and suppression of anti-apoptotic genes such as *prkce*,^10, 26^ *birc3*^10^ and *bcl2*.^10, 27^ When we tested for expression of I-IFN*s* (pan-*IFNα* and *IFNβ*) in HFFs, infected with either WT or ΔDUB-HCMV, or transfected with poly(I:C), as a known inducer only, ΔDUB-HCMV and poly(I:C), significantly increased the transcript levels of pan-*IFNα* (Figure 4a) and *IFNβ* (Figure 4b) at all tested time points, compared with WT-HCMV. Interestingly, the expression-pattern of many of the pro-apoptotic and anti-apoptotic genes aligned with the expression-pattern of I-IFN*s* by WT- and ΔDUB-HCMV (e.g., compare transcription patterns in Figures 3a and b and Figures 4a and b), which suggests that inhibition of pro-apoptotic and induction of anti-apoptotic gene expression by WT-HCMV infection, could be due to inhibition of I-IFN synthesis.

HCMV induces innate immunity through several PRRs, including endosome localized TLR9 and cytosolic DNA sensors (CDSs).^28, 29, 30, 31, 32^ TLR9 and CDSs, relay their signals through Myeloid differentiation primary response gene88 (MyD88) and STING, respectively, for the synthesis of I-IFNs.^33, 34, 35^ To understand the role of HCMV in I-IFN synthesis and its dependence on MyD88- or STING-pathway, these genes were knocked down in HFFs using short-hairpin RNA (shRNA), wherein *myd88* and *sting* transcripts were significantly reduced compared with control shRNA for GFP (Supplementary Figures S2a**–**S2b). HFFs deficient in either *myd88* or *sting* were mocked or infected with WT-HCMV for analysis of I-IFN synthesis. WT-HCMV induced the synthesis of *IFNβ* in control HFFs whereas reduced the synthesis in *mydd88* or *sting* knocked down HFFs (Figures 4c and d). This observation suggests that in absence of either MyD88 or STING, induction of I-IFN synthesis is compromised during HCMV infection, which further confirms a dependency on MyD88 and STING for I-IFN synthesis, during HCMV infection. To understand the role of HCMV-DUB (UL48N) in I-IFN synthesis, genes for MyD88 or STING were paired with UL48N and co-expressed in HEK293 cells together with the luciferase reporter gene regulated either by the *IFNα4*, *IFNα6* or *IFNβ* promoter. All I-IFN promoters increased luciferase production when co-expressed with either the MyD88 or STING gene, however, in all cases the increase was inhibited upon co-expression of UL48N. (Figures 4e–g and Supplementary Figure S2c). UL48N overexpression also inhibited CpG (TLR9-Ligand)-stimulated transcription of I-IFN and *IP10* in HeLa cells (Figures 4h and i). Furthermore, we tested the efficacy of HCMV-DUB in affecting the I-IFN-dependent anti-viral immunity by transfecting IMR32 cells with UL48N or UL48NΔDUB constructs, followed by infecting the cells with New Castle Disease Virus (NDV), 24 h post transfection (hpt). Testing for NDV-replication by quantitative (q)-PCR, showed marked increase in NDV-replication in cells transfected with UL48N but not with UL48NΔDUB (Figure 4j). This finding suggests that UL48N suppressed cellular anti-viral responses, which was further proved by analyzing the replication status of both WT and ΔDUB-HCMV in HFFs wherein replication of WT-HCMV was gradually increased with time compared with ΔDUB-HCMV (Supplementary Figure S2d).

Further, to investigate the role of HCMV-DUB in antagonizing I-IFN-induced cell death, HCMV-infected HFFs were treated with recombinant-IFNβ (rIFNβ) on third dpi. An MTT assay, done 3 days after rIFNβ treatment, showed antagonization of rIFNβ-mediated cell death by WT-HCMV-infected HFFs (Figure 4k). To further analyze I-IFN-antagonizing ability of UL48N for oncogenesis, IMR32 cells were transfected with different amounts of plasmid encoding UL48N or UL48NΔDUB and wound was created 24 hpt. Cell monolayer with wounds were treated with rIFNβ and observed after 72 h for wound healing. The UL48N-transfected IMR32 cells showed dose-dependent wound-healing compared with cells transfected with UL48NΔDUB or Vec, and cells treated with rIFNβ only (Figure 4l). Amount of FLAG-tagged UL48N and UL48NΔDUB was measured by immunoblotting (Figure 4l). Finally, to investigate a correlation of I-IFN inhibition with induction of oncogenic properties, receptor for I-IFNs (IFNAR) on HFFs was blocked using anti-IFNAR2 antibody to ultimately inhibit positive feedback signaling. Following blockade of IFNAR, HFFs were infected with WT- or ΔDUB-HCMV and 3 dpi, transcript status of *IFNβ* was analyzed that showed an inhibition upon IFNAR blockade (Figure 4m). The cell proliferation and viability status were analyzed with same cells by doing *MKi67* transcript quantification and MTT assay, respectively. Cells treated with anti-IFNAR2 antibody showed enhanced proliferation (Figure 4n) and viability (Figure 4o) compared with antibody-untreated cells that inversely correlated with *IFNβ* expression in same cells (Figures 4m and o). This suggests that induction of oncogenic properties upon WT-HCMV infection is owing to inhibition of I-IFNs. In addition, to understand the reason behind enhanced G2-phase entry of WT-HCMV-infected cells, cyclin-D1 transcript level was measured in HCMV-infected HFFs after 2 dpi, as I-IFN inhibits cyclin-D1 expression to block cell cycle at G1-phase.^36, 37, 38, 39, 40, 41, 42, 43, 44, 45^ Interestingly, cyclin-D1 (*ccnd1*) expression was upregulated upon WT-HCMV infection compared with mock or ΔDUB-HCMV infection to HFFs (Supplementary Figure S2e), which inversely correlated with the I-IFN results observed in Figures 4a and b. These data collectively suggest that HCMV-DUB could inhibit I-IFN synthesis and I-IFN-mediated death of infected cells and thus promote oncogenic properties.

### HCMV-DUB deubiquitinates cytoplasmic molecules to inhibit I-IFN synthesis and promote carcinogenic properties

**Figure 5:**
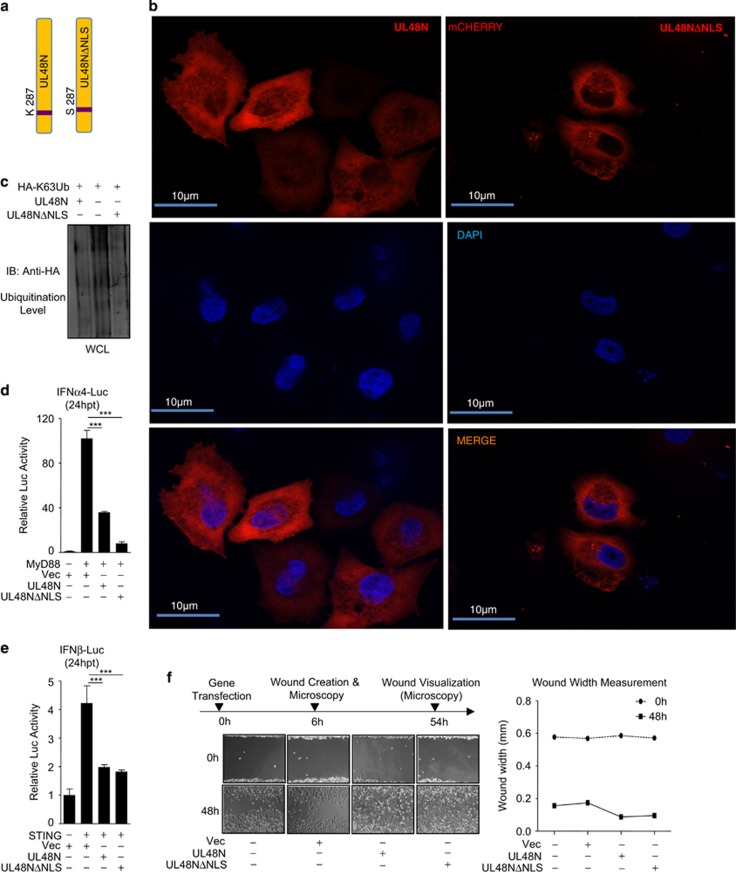
Nuclear Localization Sequence of UL48N is dispensable for UL48-DUB activity (**a**) Schematic representation of UL48N and its NLS-mutant (UL48NΔNLS). (**b**) The cellular localization of mCherry-tagged UL48N and UL48NΔNLS was observed under the fluorescence microscope at × 63. Nucleus was stained with Hoechst 33342. (**c**) DUB activity of UL48N and UL48NΔNLS was compared, by observing the ubiquitination status of whole cell lysates (WCL), upon overexpressing UL48N and UL48NΔNLS in HEK293 cells, as indicated. (**d**, **e**) Luciferase assay, was done for *IFNα4* or *IFNβ* promoter by co-transfection of MyD88 or STING and UL48N or UL48NΔNLS expression plasmids as depicted. (**f**, left, right) UL48N or UL48NΔNLS was over-expressed in IMR32 cells, wound was created and wound-healing was observed at 48 h and width of the wound was measured. Shown results are the representative of Single (**b**, **c**) or two (**d**, **e** and **f**) independent experiments. Differences were considered statistically significant with a ***P-value<0.001

The pUL48 contains nuclear localization sequences (NLS), suggesting that differential localization of pUL48N within the cell may be critical for its DUB function and I-IFN inhibition. To test this, NLS-mutant of UL48N was created as described previously^46^ and named as UL48NΔNLS (Figure 5a), which was unable to enter the nucleus (Figure 5b) and was equally efficient DUB as UL48N (Figure 5c). To analyze the function of UL48N (cytoplasmic or nuclear function) in I-IFN synthesis pathways, I-IFN promoter activity was measured with overexpression of UL48N and UL48NΔNLS. To this end, *IFNα4* and *IFNβ* promoter was activated by co-expression of MyD88 or STING, which are the key molecules of TLR9 or CDS signaling pathways, respectively. UL48N and UL48NΔNLS, both inhibited MyD88 or STING-mediated *IFNα4* or *IFNβ* promoter activity, respectively (Figures 5d and e). We hypothesized that UL48N deubiquitinates the cytoplasmic (not the nuclear) signaling molecules activated by PRRs, whose ubiquitination is necessary for induction of I-IFNs. We also compared UL48NΔNLS with UL48N in a wound-healing assay and found that wounds of IMR32-monolayers expressing either of the constructs, closed with equal efficiency after 48 h, (Figure 5f), despite a modest difference in I-IFN inhibiting property. This indicates that NLS of pUL48 is dispensable for the HCMV-DUB induced cell migration observed above (e.g., Figure 3f). These results show that nuclear localization of UL48N is not required to show inhibitory effect on I-IFN synthesis and cellular migratory activities.

### HCMV-DUB deubiquitinates PRR-mediated signal transducers to inhibit I-IFN synthesis

**Figure 6:**
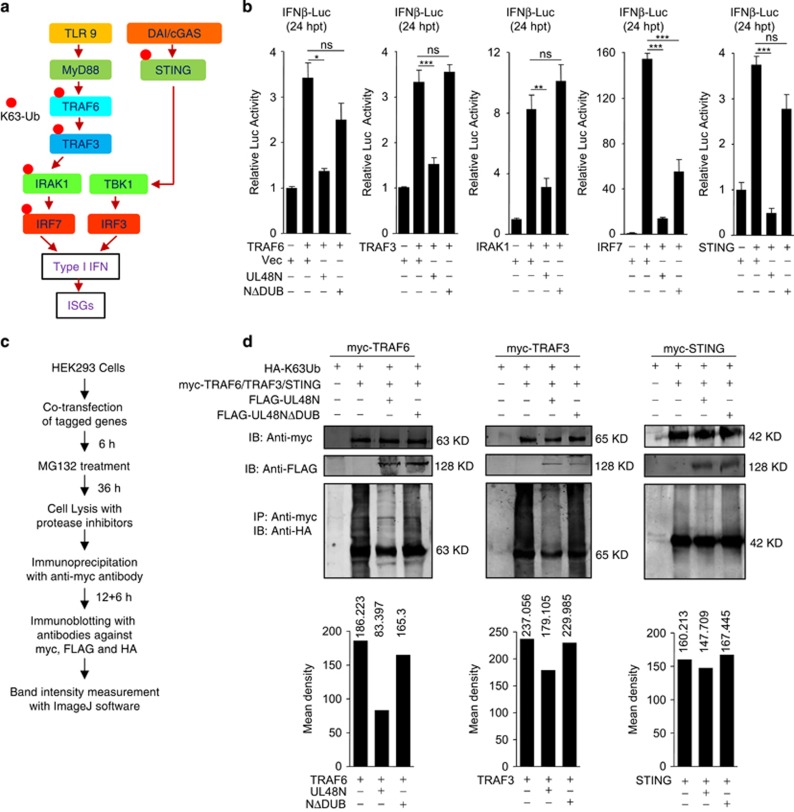
UL48N targets TRAF6, TRAF3 and STING for deubiqutination and inhibition of interferon synthesis. (**a**) Schematic representation of TLR, DNA sensing (DAI and cGAS) signaling pathways and ubiquitination of signaling molecules. (**b**) Luciferase assay was done for *IFNβ* promoter by co-transfection of TRAF6, TRAF3, IRAK1, IRF7 or STING, with UL48N or UL48NΔDUB expression plasmids as depicted. (**c**,**d**) Ubiquitination status of TRAF6, TRAF3 and STING was analyzed in the presence of overexpression of UL48N or UL48NΔDUB in HEK293 cells. Co-transfection of HA-K63Ub, myc-TRAF6, myc-TRAF3 or myc-STING with FLAG-UL48N or FLAG-UL48NΔDUB was done as depicted. (**d**, top) Immunoprecipitated sample was blotted with anti-HA, anti-myc and anti-FLAG antibody to detect respective proteins, (**d**, bottom) Density of TRAF6, TRAF3 or STING ubiquitination was calculated and compared for each sample by using ImageJ software. Luc: Luciferase, hpt: hours post transfection, Vec: Empty Vector. Shown results are the representative of three (**b**) or two (**d**) independent experiments. Differences were considered statistically significant with a *P-value<0.05, **P-value<0.01 and ***P-value<0.001, ns, non-significant difference (*P*-value>0.05). See also Supplementary Figure S3a-S3b

To facilitate the synthesis of I-IFNs through PRRs, many signaling mediators get K63 (lysine-63) ubiquitinated (Figure 6a). As K63 ubiquitination of cytoplasmic molecules is an important event during innate immune signaling, our next approach was to find out the cytoplasmic target molecule whose deubiquitination is facilitated by UL48N and is necessary for inhibition of I-IFN synthesis. To this end, various signaling molecules of the TLR and CDS pathways, undergoing K63 ubiquitination for induction of I-IFNs synthesis, were co-expressed with UL48N or UL48NΔDUB in HEK293 cells and were tested for *IFNβ* promoter activity by the Luciferase assay. Co-expression of UL48N with TRAF6, TRAF3, IRAK1, IRF7 or STING, inhibited *IFNβ* promoter activity whereas UL48NΔDUB completely rescued the inhibition in case of TRAF3 and IRAK1, and partially rescued in case of TRAF6, IRF7 and STING (Figure 6b). We concluded that UL48N is able to deubiquitinate multiple signaling molecules to inhibit I-IFN production. Finally, the K63-deubiquitination activity of UL48N was tested by immunoblot analysis using various constructs such as myc-tagged TRAF6, TRAF3, IRAK1, IRF7 or STING and FLAG-tagged UL48N and UL48NΔDUB as indicated in Figure 6c. The result showed that UL48N hugely deubiquitinates TRAF6, TRAF3 and STING (Figure 6d) and slightly deubiquitinates IRAK1 (Supplementary Figure S3a) and IRF7 (Supplementary Figure S3b). Collectively, these results suggest that HCMV-DUB targets various key molecules of the signaling cascade to suppress I-IFN and to induce oncogenesis.

## Discussion

**Figure 7:**
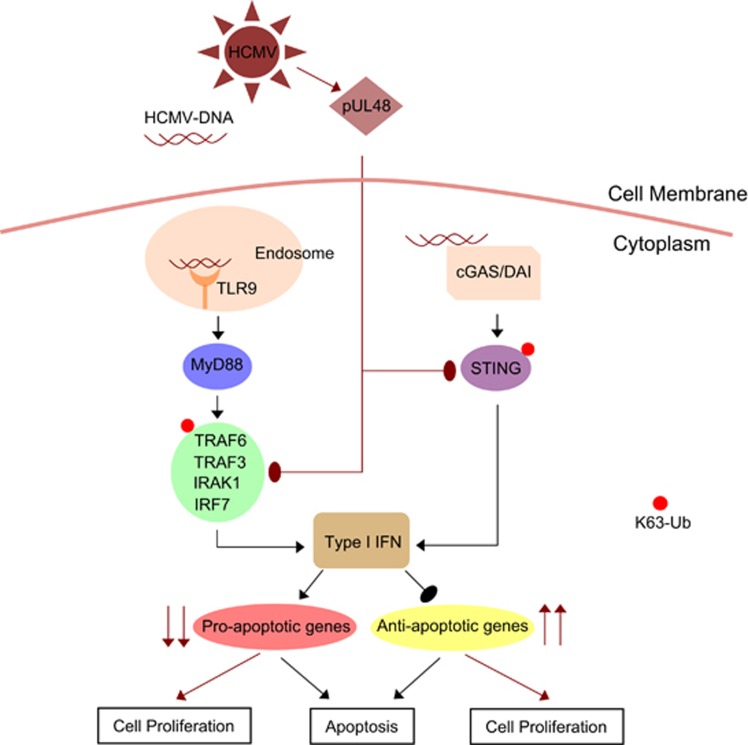
Induction of oncogenesis through inhibition of I-IFN synthesis by HCMV deubiqutinase (pUL48). Model showing the role of HCMV deubiquitinase in inhibiting I-IFN synthesis to promote oncogenesis. HCMV-DUB facilitates deubiquitination of TRAF6, TRAF3, IRAK1, IRF7 and STING to inhibit I-IFN synthesis, which in turn inhibits the expression of several pro-apoptotic genes and induces the expression of anti-apoptotic genes. Black arrows show normal cellular pathways. Maroon arrows show pathways during HCMV infection. Blunt arrows designate inhibitory action and pointed arrows designate stimulatory action. Red bubbles designate K63 ubiquitination of signaling molecules

HCMV association with various cancer tissues is well-established, however role of HCMV components in oncogenesis is poorly understood. Studies showed indirect roles of HCMV immediate-early proteins (IE1 and IE2) in oncogenesis,^47, 48, 49^ however, lacked the information on underlying molecular mechanism in oncogenesis. Our study suggests that as a consequence of overcoming anti-viral innate immune response, HCMV infection and enzymatic activity of the HCMV-DUB, in particular, may initiate early steps in oncogenesis (Figure 7). This conclusion is based on the findings stemming from HCMV-infected cells and extended to transfection experiments done with the HCMV-DUB and specific cellular anti-viral response molecules. We showed that WT-HCMV infection to non-transformed HFFs, increased cell metabolic activity, cell proliferation, promoted rapid entry into G2-phase of cell cycle, resistance to apoptotic stimuli, enhanced anti-apoptotic gene expression (*bcl2*, *birc3*, *prkce*, *survivin*, *xiap*), and reduced expression of several pro-apoptotic gene (*fadd*, *caspase-8*, *p21*, *tnfα*) compared with ΔDUB-HCMV infection. The expression level of several other anti-apoptotic genes (*ciap1*, *cflip*, *bcl-xl*, *mcl-1* and *hif1α*) was either reduced in WT-HCMV-infected HFFs or was comparable to ΔDUB-HCMV infection. Similarly, we observed that expression level of several other pro-apoptotic genes (*bad* and *bax*, *bak*, *noxa*, *puma*) was either reduced or remained unchanged due to infection with both, WT- and ΔDUB-HCMV in HFFs. These observations suggest that differential expression-pattern of anti- or pro-apoptotic genes might be associated with stimuli, stimuli-duration or/and cell type. In addition, it may be possible that in other cell type various other anti- or pro-apoptotic genes might also be involved in the cell survival signaling upon HCMV infection. Collectively, we observed majority of anti- and pro-apoptotic genes are affected due to HCMV infection in HFFs to protect cells from apoptosis as well as to bring about oncogenesis via overall promoting cell survival and carcinogenic properties.

To understand the molecular mechanism of these changes, the UL48-DUB domain was expressed in IMR32 cell line, wherein its ability to influence other characteristics of cancer cells such as proliferation, migration, glucose uptake and tissue invasion could be studied more directly. Previous studies have also shown HCMV-infected human cells to be resistant to apoptosis as well as uptake more glucose, but with little evidences of tumorigenesis or mechanisms.^47, 49, 50, 51, 52^

Several studies show cross-talk between oncogenesis and innate immunity wherein I-IFNs promote apoptosis via expression of pro-apoptotic genes^10, 22^ and inhibition of anti-apoptotic genes.^10^ Other studies suggest that overexpression of anti-apoptotic genes like *bcl2* protects cells from I-IFN-mediated cell death.^27, 53^ In our study, we show WT-HCMV-infected cells express reduced *I-IFNs* compared with ΔDUB-HCMV-infected cells, which confirms HCMV-DUB as a key inhibitor of I-IFNs sythesis, that might involve in induction of oncogenic properties in HFFs. We have also shown that UL48-DUB inhibits I-IFN synthesis induced through different PRRs-mediated signaling pathways wherein UL48-DUB deubiquitnates signaling mediators such as TRAF6, TRAF3, IRAK1, IRF7 and STING. Interestingly, in case of TRAF6, IRF7 and STING-mediated induction of I-IFN, NΔDUB-UL48 showed a partial repression, which also suggests a DUB-independent role of UL48N in oncogenesis. As HCMV inhibits I-IFN to counteract host innate immunity,^54, 55^ inhibition of I-IFN synthesis by HCMV indicates its potential to establish a pro-cancer microenvironment. In addition, studies suggest that people having a particular SNP in TLR9 (2848GA) are more susceptible to HCMV infection and cancer development.^29, 56^ Here, we have found evidence for hypothetical dual role for HCMV-DUB, in defeating host anti-viral responses and also promoting an oncogenic state.

Several viruses encode DUBs and most of these interfere with innate immune signaling pathways.^57, 58, 59^ Our findings rationalize that similar to HCMV, other DUB-encoding viruses may perturb normal cell cycle or apoptotic pathways by inhibiting I-IFN synthesis.^58, 60^ Hence, our study provides new insight into mechanisms of virus-induced oncogenicity related to inhibition of innate immunity. Collectively, our study suggests that HCMV could be a factor leading to development of cancer through UL48-DUB in immunocompromised or immune-declined aged individuals and HCMV-DUB could be a potential therapeutic target to manage HCMV-associated cancers.

## Materials and methods

### Cell lines, plasmids, antibodies and viruses

Human Embryonic Kidney (HEK293) and HeLa cell lines were obtained from American Tissue Culture Collection (ATCC). Neuroblastoma IMR32 cell line was obtained from National Centre for Cell Science (NCCS) Pune, India and HFFs were obtained from Professor Wade Gibson’s Lab, Johns Hopkins, School of Medicine. All cells were cultured in Dulbecco’s Modified Eagle’s Medium (DMEM) supplemented with 10% FBS and 1 × Anti-Biotic Anti-Mycotic, supplied by Invitrogen (Carlsbad, CA, USA) by Thermo Fisher Scientific (Waltham, MA, USA).

N-terminal of UL48 (UL48N, 1–1162 amino acids) was cloned in FLAG-tagged mammalian expression vector pCMV3Tag1a. UL48NΔDUB (a double mutant of C24I and H162A) UL48NΔNLS (a single mutant of K287S) were created by using UL48N as template and cloned in pCMV3Tag1a. IRAK1, TRAF3, TRAF6, MyD88, STING were cloned in myc-tagged mammalian expression vector pCMV3Tag2a. Plasmids containing Firefly Luciferase gene under IFN*β*, IFN*α*4 and IFN*α*6, ISRE promoters, and Myc-tagged IRF7 were obtained from Professor Shizuo Akira’s (Osaka University, Japan). HCMV genomic DNA, pCMV3Tag1a (FLAG), pCMV3Tag2a (myc) and pRL-TK were kind gifts from Professor Yan Yuan, University of Pensylvania, Philadelphia. The p21promoter-Luciferase construct was obtained from Professor Martin Walsh’s Laboratory through Addgene (Cambridge, MA, USA).

Mouse raised anti-FLAG, Rabbit raised anti-myc and Rabbit raised anti-HA antibody were purchased from Sigma Aldrich (St. Louis, MO, USA). Mouse raised anti-myc antibody was purchased from Invitrogen by Thermo Fisher Scientific. IR dye labeled anti-Rabbit and anti-Mouse IgG (secondary antibody), were purchased from LI-COR. Rabbit raised anti-p53 were purchased from Invitrogen. Rabbit raised anti-p21, anti-BCL2, anti-Caspase-3, anti-Survivin were purchased from Cell Signaling Technology (Danvers, MA, USA), anti-BIRC3 was purchased from Santa Cruz Biotechnology (Dallas, TX, USA).

GFP-tagged (strain AD169), wild-type (H-WT) and H162A mutant (HΔDUB, or DUB-mutant WT-HCMV and DUB-mutant HCMV were kind gifts from Professor Wade Gibson, The Johns Hopkins University School of Medicine. AD169 is extensively passaged, fibroblast adapted laboratory strain of HCMV, which is non-pathogenic; NDV-GFP was a kind gift from Professor Peter Palese, Icahn School of Medicine at Mount Sinai.

### Virus infection

HFFs were grown to full confluence and infected with GFP-tagged HCMV at 5 MOI. At 24 h post infection, virus infection (% infection) was measured through flow cytometer for FITC (GFP-HCMV). Six days post infection, CPE was observed and cells were harvested for RNA isolation. For cell cycle, Ki67 staining and apoptosis study, infection was done for three and four days respectively. IMR32 cells were infected with NDV-GFP (5 MOI) in serum free DMEM for 1 h. Cells were then grown in reduced serum (1% FBS) DMEM for 36 h.

### MTT assay

MTT [3-(4,5-dimethylthiazol-2-yl)-2,5- diphenyltetrazolium bromide] assay was done as described previously.^10^

### Quantitative RT PCR

Designated cells were either infected with virus or transfected with respectively designated plasmids containing UL48N, UL48NΔDUB (1 *μ*g/well, 12-well plate), or 1 *μ*g/well (24-well plate) of p(I:C), or stimulated with CpG (0.5 *μ*M/well) for indicated times. Total RNA was isolated using Trizol reagent (Ambion/Invitrogen) and cDNA was synthesized using iScript cDNA synthesis kit (BioRad, Hercules, CA, USA) as per the manufacturer’s protocol. cDNA was used to analyze the transcript level in samples as indicated. Real time quantification was done using StepOne Plus Real time PCR Systems by Applied BioSystems (Foster City, CA, USA). Primers used for quantitative RT PCR are previously mentioned^10^ and also listed in Supplementary Table1.

### Cell cycle flowcytometry

HFFs were grown to 100% confluence and infected with wild-type and DUB-mutant HCMV. After 3 days of infection cells were harvested and fixed with 70% chilled ethanol. RNaseA (5 *μ*l of 10 mg/ml stock) was added to ethanol and incubated at 4 °C for 30 mins. Cells were washed with 1 × PBS and propidium iodide (PI, 10 *μ*l of 100 mg/ml stock) was added to cells and incubated Overnight at 4 °C. PI-based cell cycle flow cytometric analysis was done using FACS Aria III (Becton Dickinson, Franklin Lakes, NJ, USA) and data were analyzed by using FlowJo software version 10 (FlowJo, Ashland, OR, USA).

### Annexin-PE flowcytometry

HFFs were grown to 100% confluence and infected with wild-type and DUB-mutant HCMV. On second dpi, cells were treated with etoposide (30 *μ*M) and incubated for 24 h. On third dpi cell were trypsinised, harvested, washed with 1 × PBS and processed for Annexin-PE Flow Cytometery using the BD Biosciences (Haryana, India) Annexin V apoptosis kit (Cat No# 556422) as per the manufacturer’s protocol. Flowcytometric analysis was done using FACS Aria III (Becton Dickinson) and data were analyzed by using FlowJo software version 10 (FlowJo).

### MKi67 staining

HCMV-infected HFFs were harvested on 3 dpi, washed with 1 × PBS, fixed with 70% chilled ethanol at −20 ^o^C for 2 h. Cells were washed with staining buffer (1%FBS and 0.09% NaN3 made in 1 × PBS). 1:200 dilution of APC labeled Ki67 antibody (Novus Biologicals, Littleton, CO, USA, Cat No.# NB110-89717APC) was used for staining the fixed cells at room temperature. Stained cells were analyzed for MKi67 nuclear staining using FACS Aria III (Becton Dickinson) and data were analyzed by using FlowJo software version 10 (FlowJo).

### MKi67 stable cell generation and HFF knockdown

UL48N and UL48NΔDUB constructs were prepared by subcloning into pMSCV-puro vector. Transduction of IMR32 cells was performed as described earlier (Tailor *et al.* 2007). In brief, viral supernatants were prepared in BOSC23 packaging cell line using construct for VSV-G. Cells were spinoculated (2400 rpm, 33 °C, 1 h) with viral supernatants and selected using 0.2 *μ*g/ml of Puromycin for 48 h.

Knockdown experiment: HFFs were transfected with shRNA of MyD88 (Clone D1) or STING (Clone A8), obtained from Sigma Aldrich and selected on puromycin. The transcript level of *myd88* and *sting* was analyzed through qPCR. HCMV infection (5 MOI) was done to knockdown cells for 3 days and on 3 dpi cells were harvested to isolate RNA for real time analysis of *IFNβ* transcripts. The shRNA against GFP (sh005) was taken as knockdown control.

### Transfection

Transfection was done using Lipofectamine 2000 or 3000 (Invitrogen) as per the manufacturer’s protocol. For transfection related to immunoprecipitation, after 6–7 h post transfection, MG132 was added to the transfected cells to a final concentration of 0.5 *μ*M and incubated for further 30–40 h.

### Glucose uptake assay

Glucose uptake assay was done as previously described.^61^ Cells were seeded at a density of 0.1 x 10^6^ cells/well into 48-well plates in 200 *μ*l culture medium and incubated for 24 h. Cells were then incubated in serum and glucose free DMEM for 2 h. Serum and Glucose starved cells were treated with 10 *μ*M 2-NBDG (Invitrogen) for 10 min at 37 °C and then analyzed on BD FACS CantoII flow cytometer. 2-NBDG fluorescence was detected on FITC channel. 2-NBDG: (2-(N-(7-Nitrobenz-2 oxa-1,3-diazol-4-yl)Amino)-2-Deoxyglucose.

### Luciferase based promoter Assay

HEK293 cells were transfected with empty vector, expression plasmids containing genes for either, MyD88, STING, TRAF6, TRAF3, IRAK1 or IRF7 (250 ng/well, 24-well plate), UL48N (250 ng/well, 24-well plate), UL48NΔDUB or UL48NΔNLS (250 ng/well, 24-well plate), along with 100 ng of IFN*α*4, IFN*α*6, IFN*β* and ISRE promoter containing Firefly luciferase expression plasmid and Renilla Luciferase containing plasmids (pRL-TK, 5 ng/well) as depicted. Luciferase assay was done with total cell lysate containing above-mentioned promoters and gene constructs by using Dual-Glo, Luciferase assay system (Promega, Madison, WI, USA) as per the manufacturer’s protocol. Luminescence of each sample was measured by the Glomax (Promega).

### Wound-healing assay

IMR32 Cells were grown at 100% confluence and transfected with designated plasmids (1 *μ*g/well, 12-well plate, or as indicated). A sharp wound was created using 200 μl pipette tips, 6 h post transfection. Wounds were visualized at × 10 Sigma Aldrich objective lens under the bright field microscope and three random images were captured for few days as indicated. Wound width was measured and plotted as a line graph using GraphPad Prism Version 5.

### Matrigel invasion assay

Matrigel invasion assay was done as demonstrated at http://www.abnova.com

### Immunoprecipitation (IP) and Immunoblotting

For deubiquitination studies, each plasmid was taken 2 *μ*g in concentration for transfection into cells grown in 35 mm culture dish. Cells were treated with 0.5 *μ*M MG132, 6 h post transfection. Cells were harvested after 36 h. of transfection with standard cell lysis buffer supplemented with 1 × protease inhibitor cocktail (obtained from Sigma Aldrich) and 10 mM NEM (*N*-Ethyl Maleimide). Immunoprecipitation and Immunoblotting were done as described previously.^62^ Immunoblotted nitrocellulose membrane was imaged with LI-COR system.

For endogenous protein detection, mock or HCMV-infected HFFs (grown in six-well plate) were lysed in 30 *μ*l of standard cell cell lysis buffer and ~8 *μ*g of protein was loaded to each well.

### ImageJ image analysis

Western blot densitometry: an area of interest was selected on the smeary part above the protein bands shown in the immunoblots. Mean density of the selected area was then calculated.

### Microscopy

The mCherry-tagged *UL48N* and *UL48NΔNLS* were transfected in the HeLa cells. After 24 h of transfection, cells were fixed and stained with DNA staining reagent Hoechst 33342. Localization of pUL48 and pUL48NΔNLS was visualized at   × 63 with Apotome-AXIO fluorescence microscope by Zeiss. HCMV infection (GFP fluorescence) was visualized with Inverted microscope Vert.A1 (AXIO) by Zeiss.

### I-IFN receptor blocking

The IFNAR*α/β* receptor was blocked as described previously,^63^ using anti-IFNAR chain2 antibody (Clone#MMHAR2, Cat# MAB1155) from Merck. Upon blocking for 4 h, HFFs were infected with HCMV.

### Statistical analysis

Statsitical analysis was done with the help of GraphPad Prism, version 5. In the bar graph, differences between two groups were compared using an unpaired two-tailed Student’s *t*-test, whereas the differences between three or more groups was calculated by using one-way analysis of variance by Newman–Keuls test. Differences were considered statistically significant with a *P-value<0.05, **P-value<0.01 and ***P-value<0.001, ns, non-significant difference (*P*-value>0.05).
