# Supplementary material for: Essential role of HCMV deubiquitinase in promoting oncogenesis by targeting anti-viral innate immune signaling pathways
Source: Cell Death Dis. 2017 Oct 5;8(10):e3078–. doi: 10.1038/cddis.2017.461 (PMC5680583; doi:10.1038/cddis.2017.461)
Supplement: Supplementary Table 1 [file cddis2017461x1.docx]

|  | Gene | Primer Sequence (5’-3’) |
| --- | --- | --- |
| 1 | Survivin | Sense: AGAACTGGCCCTTCTTGGAGG  Anti-Sense: CTTTTTATGTTCCTCTATGGGGTC |
| 2 | XIAP | Sense: TTTGCCTTAGACAGGCCATC  Anti-Sense: TTTCCACCACAACAAAAGCA |
| 3 | cIAP1 | Sense: AGCTAGTCTGGGATCCACCTC  Anti-Sense: GGGGTTAGTCCTCGATGAAG |
| 4 | cFLIP | Sense: ATGTCTGCTGAAGTCATCC  Anti-Sense: ATCCTCACCAATCTCCTGCC |
| 5 | Mcl-1 | Sense: TGAAATCGTTGTCTCGAGTGATG  Anti-Sense: TCACAA TCGCCCCAGTTT |
| 6 | Bcl-xL | Sense: GATCCCCATGGCAGCAGTAAAGCAAG  Anti-Sense: CCCCATCCCGGAAGAGTTCATTCACT |
| 7 | BAK | Sense: CATCAACCGACGCTATGACTC  Anti-Sense: GTCAGGCCATGCTGGTAGAC |
| 8 | Bcl2 | Sense: GGTGGGGTCATGTGTGTGG  Anti-Sense: CGGTTCAGGTACTCAGTCATCC |
| 9 | BIRC3 | Sense: TTTCCGTGGCTCTTATTCAAACT  Anti-Sense: GCACAGTGGTAGGAACTTCTCAT |
| 10 | PRKCE | Sense: CAACGGACGCAAGATCGAG  Anti-Sense: CTGGCTCCAGATCAATCCAGT |
| 11 | FADD | Sense: TCTCCAATCTTTCCCCACAT  Anti-Sense: GAGCTGCTCGCCTCCCT |
| 12 | p53 | Sense: GCCATCTACAAGCAGTCACAG  Anti-Sense: TCATCCAAATACTCCACACGC |
| 13 | BAX | Sense: TCCCCCCGAGAGGTCTTTT  Anti-Sense: CGGCCCCAGTTGAAGTTG |
| 14 | BAD | Sense: CCCAGAGTTTGAGCCGAGTG  Anti-Sense: CCCATCCCTTCGTCGTCCT |
| 15 | Caspase-8 | Sense: AGAGTCTGTGCCCAAATCAAC  Anti-Sense: GCTGCTTCTCTCTTTGCTGAA |
| 16 | p21 | Sense:TGGAGACTCTCAGGGTCGAAA  Anti-Sense: GGCGTTTGGAGTGGTAGAAATC |
| 17 | 18S | Sense: ATCACCATTATGCAGAATCCACG  Anti-Sense: GACCTGGCTGTATTTTCCATCC |

**Supplementary Table1: Primer sequence for quantitative PCR**
