# Supplementary material for: Essential role of HCMV deubiquitinase in promoting oncogenesis by targeting anti-viral innate immune signaling pathways
Source: Cell Death Dis. 2017 Oct 5;8(10):e3078–. doi: 10.1038/cddis.2017.461 (PMC5680583; doi:10.1038/cddis.2017.461)
Supplement: Supplementary Figures [file cddis2017461x2.ppt]

## Slide 1
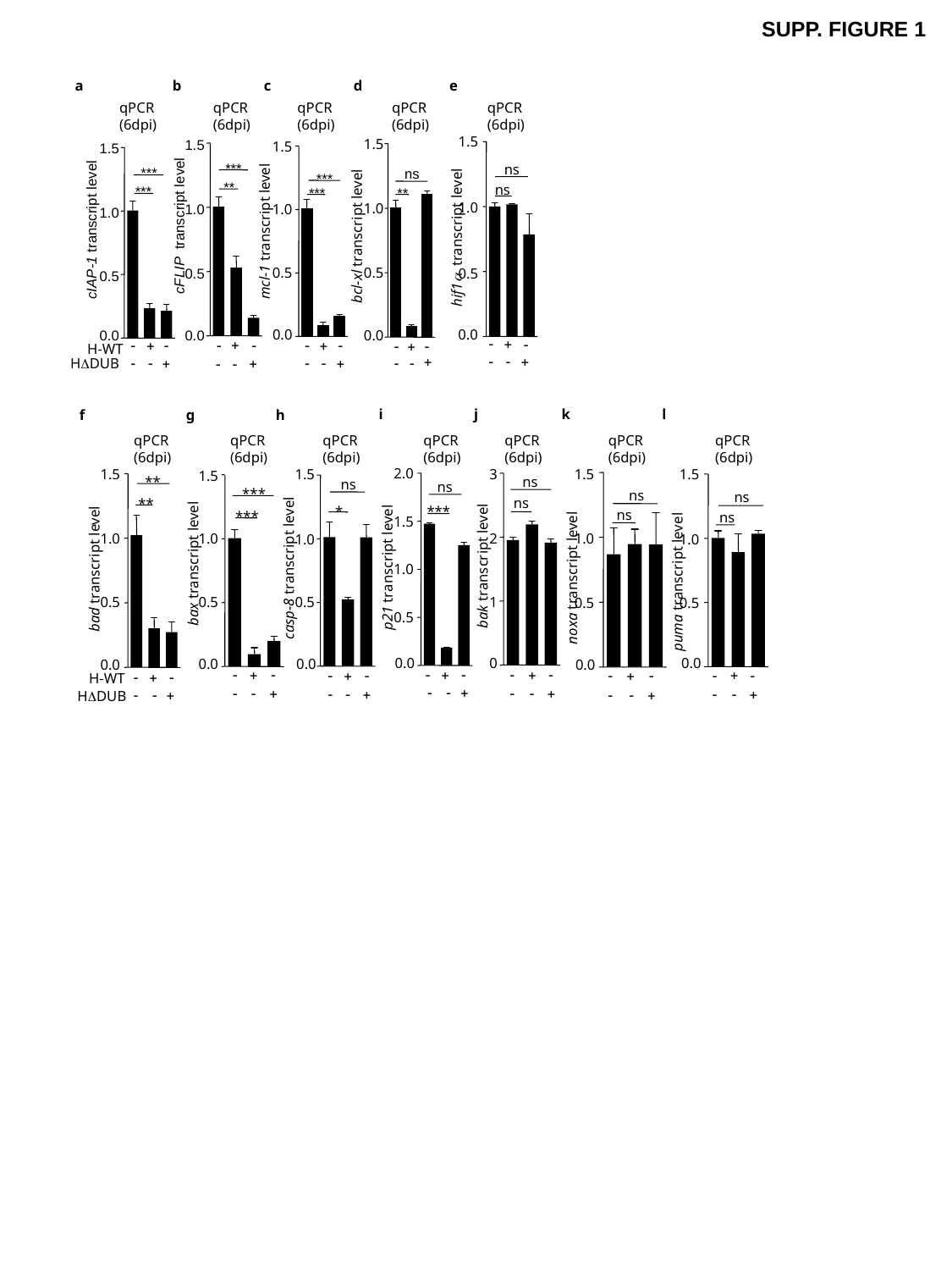

SUPP. FIGURE 1
a
b
c
d
e
qPCR
(6dpi)
qPCR
(6dpi)
1.5
1.0
cFLIP transcript level
0.5
0.0
***
**
+
−
−
+
−
−
qPCR
(6dpi)
1.5
1.0
mcl-1 transcript level
0.5
0.0
***
−
+
−
−
−
+
***
qPCR
(6dpi)
1.5
1.0
bcl-xl transcript level
0.5
0.0
ns
**
−
+
−
+
−
−
qPCR
(6dpi)
1.5
1.0
hif1α transcript level
0.5
0.0
−
+
−
+
−
−
ns
ns
1.5
1.0
cIAP-1 transcript level
0.5
0.0
***
***
H-WT
HΔDUB
−
+
−
−
−
+
i
j
k
l
f
g
h
qPCR
(6dpi)
qPCR
(6dpi)
qPCR
(6dpi)
qPCR
(6dpi)
qPCR
(6dpi)
qPCR
(6dpi)
qPCR
(6dpi)
2.0
1.5
p21 transcript level
1.0
0.5
0.0
ns
***
−
+
−
−
−
+
3
2
bak transcript level
1
0
ns
ns
−
+
−
−
−
+
1.5
1.0
puma transcript level
0.5
0.0
+
−
−
−
−
+
ns
ns
1.5
1.0
bad transcript level
0.5
0.0
**
**
−
+
−
−
−
+
1.5
1.0
noxa transcript level
0.5
0.0
−
+
−
−
−
+
ns
ns
1.5
1.0
casp-8 transcript level
0.5
0.0
ns
*
−
+
−
−
−
+
1.5
1.0
bax transcript level
0.5
0.0
***
***
−
+
−
−
−
+
H-WT
HΔDUB

## Slide 2
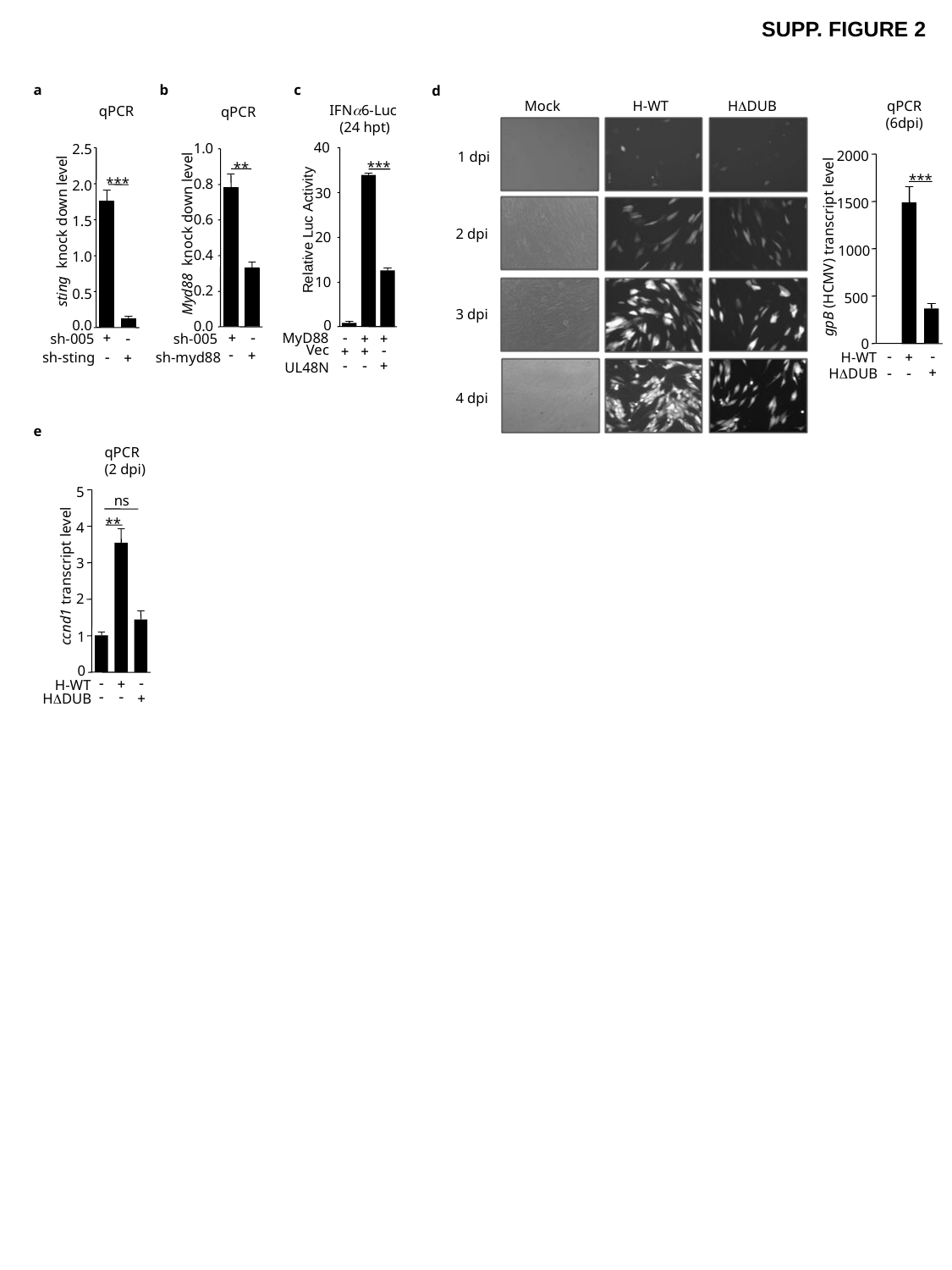

SUPP. FIGURE 2
a
b
c
d
Mock
H-WT
HΔDUB
 1 dpi
2 dpi
3 dpi
4 dpi
qPCR
(6dpi)
2000
***
1500
gpB (HCMV) transcript level
1000
500
0
−
+
−
H-WT
+
−
−
HΔDUB
qPCR
qPCR
IFNα6-Luc
(24 hpt)
40
***
30
20
10
0
−
+
+
+
+
−
+
−
−
MyD88
Vec
UL48N
Relative Luc Activity
1.0
0.8
0.6
Myd88 knock down level
0.4
0.2
0.0
**
+
−
−
+
sh-005
sh-myd88
2.5
2.0
1.5
sting knock down level
1.0
0.5
0.0
***
+
−
+
−
sh-005
sh-sting
e
qPCR
(2 dpi)
5
ns
**
4
3
ccnd1 transcript level
2
1
0
−
+
−
H-WT
−
−
+
HΔDUB

## Slide 3
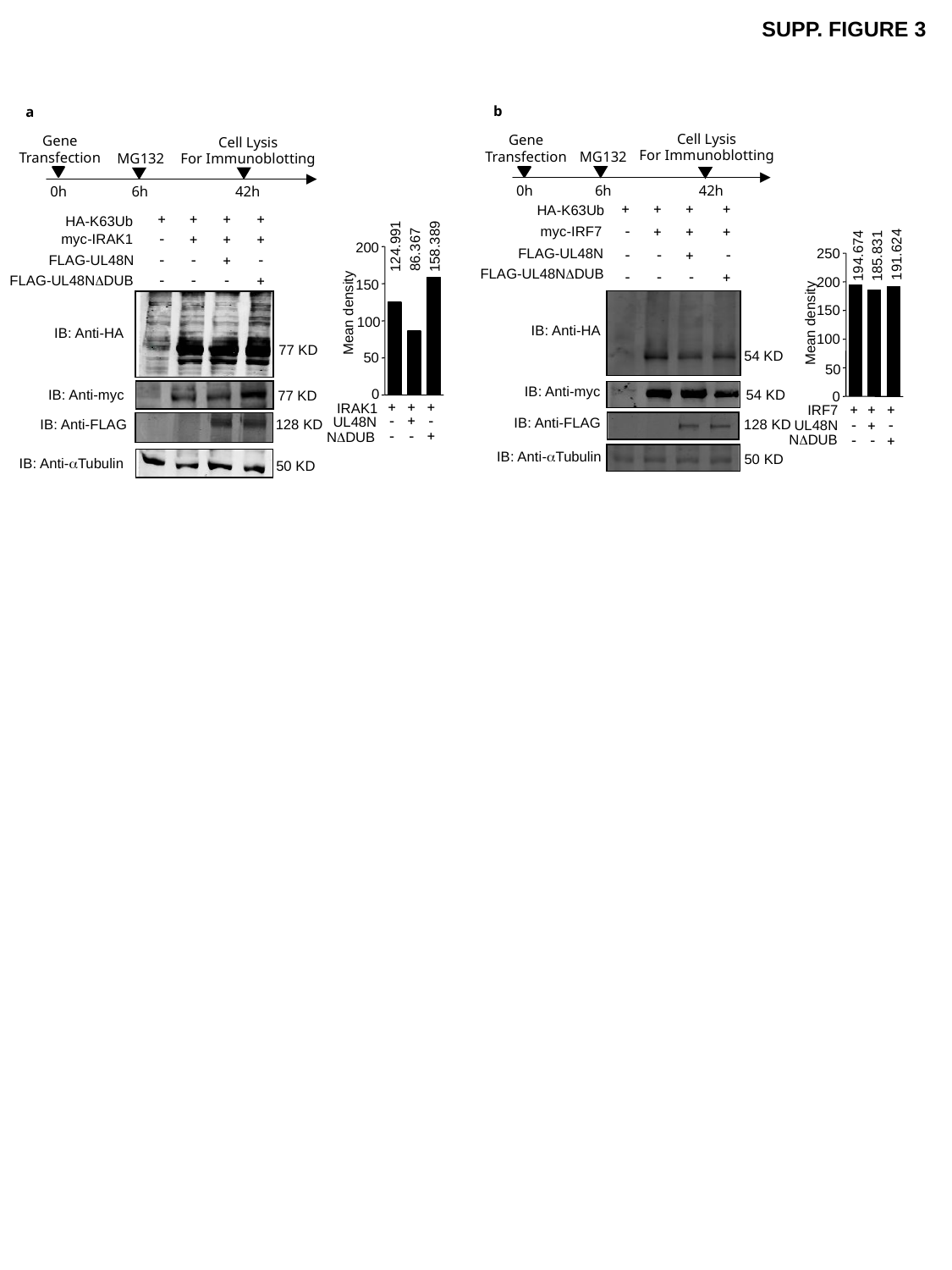

SUPP. FIGURE 3
b
a
Cell Lysis
For Immunoblotting
Gene
Transfection
0h
42h
MG132
6h
Gene
Transfection
0h
Cell Lysis
For Immunoblotting
42h
MG132
6h
+
+
+
+
−
+
+
+
−
−
+
−
−
−
−
+
HA-K63Ub
myc-IRF7
FLAG-UL48N
FLAG-UL48NΔDUB
IB: Anti-HA
IB: Anti-myc
IB: Anti-αTubulin
IB: Anti-FLAG
54 KD
54 KD
128 KD
50 KD
+
+
+
+
−
+
+
+
−
−
+
−
−
−
−
+
HA-K63Ub
myc-IRAK1
FLAG-UL48N
FLAG-UL48NΔDUB
IB: Anti-HA
IB: Anti-myc
IB: Anti-αTubulin
77 KD
77 KD
IB: Anti-FLAG
128 KD
50 KD
124.991
158.389
86.367
200
150
Mean density
100
50
0
+
+
+
IRAK1
UL48N
NΔDUB
+
−
−
+
−
−
191.624
194.674
185.831
250
200
150
Mean density
100
50
0
+
+
+
IRF7
UL48N
−
+
−
NΔDUB
−
−
+
