# Supplementary material for: Essential role of HCMV deubiquitinase in promoting oncogenesis by targeting anti-viral innate immune signaling pathways
Source: Cell Death Dis. 2017 Oct 5;8(10):e3078–. doi: 10.1038/cddis.2017.461 (PMC5680583; doi:10.1038/cddis.2017.461)
Supplement: Supplementary Figure Legends [file cddis2017461x3.doc]

**SUPPLEMENTARY LEGENDS**

**Figure S1a-S1l:** HFFs were infected with equal MOI (5 MOI) of GFP-tagged WT-HCMV (H-WT) and ΔDUB-HCMV (HΔDUB) for 6 days and 6 dpi analyzed for (**a-e**) anti-apoptotic genes *ciap1*, *cflip*, *bcl-xl, mcl1* and *hif1α* and (**f-l**) pro-apoptotic genes *bad*, *bax*, *caspase-8*, *p21*,, *bak*, *noxa* and *puma* apoptosis-associated genes after 6 dpi, by qPCR in uninfected, H-WT infected and HΔDUB HFFs. dpi: days post-infection. Shown results are the representative of three independent experiments. Differences were considered statistically significant with a *p-value<0.05, **p-value<0.01 and ***p-value<0.001, ns, non-significant difference (p-value>0.05).

**Figure S2:** Knock-down of (**a**) *myd88* and (**b**) *sting* gene was done with short-hairpin RNA. (**c**) Luciferase assay was done for *IFNα6 *promoters in HEK293 cells, as depicted. (**d, Left**) GFP-tagged HCMV-replication status was observed from day 1 to day 4 post infection in HFFs and (**d, Right**) transcript level of HCMV glycoprotein B (gpB) was analyzed by qPCR. (**e**) Transcript level of cyclin-D1 was analyzed by qPCR in HCMV infected-HFFs. dpi: days post-infection. Shown results are the representative of two (**a, b**) and three (**c-e**) independent experiments. Differences were considered statistically significant with a **p-value<0.01 and ***p-value<0.001.

**Figure S3a, S3b:** Ubiquitination status of IRAK1 and IRF7 was analyzed in the presence of overexpression of UL48N or UL48NΔDUB in HEK293 cells. Co-transfection of HA-K63Ub, myc-IRAK1or myc-IRF7 with FLAG-UL48N or FLAG-UL48NΔDUB was done as depicted.  **(a, b, Left**) Cell-lysate was blotted with anti-HA and anti-myc antibody to detect respective proteins. Alpha-tubulin was detected as loading control. (**a, b, Right**) Density of IRAK1 and IRF7 ubiquitination was calculated for each sample by using ImageJ software and compared.
